# The effect of HIIT on body composition, cardiovascular fitness, psychological well-being, and executive function of overweight/obese female young adults

**DOI:** 10.3389/fpsyg.2022.1095328

**Published:** 2023-01-18

**Authors:** Linxuan Guo, Jiaying Chen, Wenxue Yuan

**Affiliations:** School of Kinesiology and Health Promotion, Dalian University of Technology, Dalian, China

**Keywords:** HIIT, female young adults, body composition, psychological well-being, executive function

## Abstract

**Purpose:**

To evaluate the effect of a short-term HIIT program on the selected health-related parameters for overweight/obese young adult women in a university context.

**Methods:**

A total of 48 participants were randomly divided into two groups. The exercise group (HIIT) received a HIIT intervention of aerobics for 4 weeks, while the control group (CON) received no training. Body composition including waist circumference (WC), body fat percentage (BF %), Cardiorespiratory fitness (VO2max), the score of Self-Rating Depression Scale (SDS), and Stroop word-color test (SCWT) results were assessed before and after the intervention along with within- and between-group comparisons.

**Results:**

All the indices were significantly improved in HIIT group (*p* < 0.01) after 4 weeks of intervention. No significant changes were found in CON. There were significant differences between HIIT and CON in cardiovascular fitness (*p* < 0.01), SDS (*p* < 0.01) and SCWT (*p* < 0.05) before and after 4 weeks. In addition, weekly measurements of HIIT effects showed significant changes (*p* < 0.01) from the second week in the variables of body composition, VO2max, SDS and SCWT when compared with the baseline and maintained the tendency till the end of program.

**Conclusion:**

The short-term HIIT aerobics of the campus program conducted in a non-lab setting induced significant improvements in body composition, cardiovascular fitness, psychological well-being and executive function in overweight young female adults.

## Introduction

The current obesity rate for all ages in all countries shows a sustained growth trend (Wu and Hao, 2021). Obesity can cause great harm to the physical and mental health of individuals including diabetes, heart disease, stroke, arthritis, dementia, etc (Gungor, 2014), and is strongly associated with psychosocial complications such as eating disorders, deterioration of social relations, anxiety and depression (Zhong et al., 2018). In China, the overweight rate of adults ≥18 years of age was 30.1% in 2015, and the obesity rate was 11.9%, an increase of 7.3 and 4.8% from 2002, respectively (National Bureau of Disease Control and Prevention National Health Commission, 2020). The rate of overweight and obese university students in China is constantly rising (Zhang et al., 2016), with significantly higher incidence among female than male cohorts due to higher prevalence of physical inactivity in female young adults (Gungor, 2014). Image anxiety is more common in women with obesity generally, along with other mental disorders (Zhong et al., 2018). Due to estrogen, the accumulation of subcutaneous fat is more common in women, eventually developing into “peripheral obesity.” It has been found that young women are more prone to negative emotions because of their body shape (Kwan et al., 2012).

Many university students find it challenging to continue the physical exercise habits they developed during adolescence when transitioning into adulthood, due to the pressure connected with academic pursuits, work commitments, and social life (Trost et al., 2002). Young adults who generally had favorable views toward physical activity with great intentions of regular participation, often cannot follow through with their original plans made on entering university (Kwan et al., 2012). “Lack of time” is typically the biggest deterrent to regular exercise participation (Trost et al., 2002), thus a more time-effective method of exercise training has been developed. HIIT, high intensity interval training, has begun to attract the attention of researchers and the general public and has ranked among the top five training methods in the global fitness survey from 2014 to 2022. HIIT is defined as brief, intermittent bursts of vigorous activity (usually involving <100% [70–90%] of the VO_2_peak or 85–95% of the peak heart rate) interspersed with active or passive rest periods (Ito, 2019). An increasing amount of research demonstrates the effectiveness of HIIT for enhancing outcomes linked to physical and psychological health (Costigan et al., 2015). The fundamental benefit of HIIT is that it takes less time to complete than regular aerobic exercise while producing similar physiological changes and improvements in fitness and mental health (Ito, 2019).

In light of previous studies, it has been found that both active male subjects and inactive subjects of normal weight enjoy and adhere to HIIT protocols (Bartlett et al., 2011; Jung et al., 2014), but some researchers have argued that for largely inactive and/or obese individuals, the strenuous nature of HIIT may produce negative emotions toward exercise adherence and is likely to be a deterrent to participation (Bartlett et al., 2011). Inactive women were found to be unsuitable for and not benefit from this type of HIIT exercise (Kong et al., 2016). Additionally, while some studies indicate that HIIT can improve the body composition (Xi and Zhang, 2016), cardiopulmonary fitness (Li et al., 2021), emotional well-being (Strassnig et al., 2015; Korman et al., 2020; Koyuncuoğlu et al., 2021), and cognitive ability (Eather et al., 2019) of obese adults, the findings are inconsistent (Heggelund et al., 2011) and dose–response relationship of health/fitness adaptations to HIIT was still vague (Abdel-Baki et al., 2013). As a result, more empirical evidence is required to supplement available data. The purpose of this study was mainly to evaluate the effect of a short-term HIIT program on the selected health-related parameters for overweight/obese young adult women in a university context.

## Materials and methods

### Participants

The Ethical Committee of Dalian University of Technology (DLUT) for Biological and Medical Research provided ethical approval of the study (DUTSKHP220813_02). Female students at DLUT aged 18–25 years, with no existing medical conditions or injuries preventing engaging in physical activity, were recruited to participate in the intervention *via* WeChat (social media used widely in China) and DLUT Website. 52 volunteers were selected after screening. The inclusion criteria for participation were as follows: (1) non-smokers; (2) body mass index (BMI) ≥ 25 kg/m^2^ or body fat percentage (BF%) ≥ 30; (3) body weight remained constant (±2 kg) during the past 3 months; (4) no participation in any regular physical activities or exercise training; and (5) no history of metabolic, hormonal, orthopedic, or cardiovascular diseases and no current use of prescribed medication. All the potential participants were required to complete a PAR-Q form and a medical history questionnaire for further eligibility screening. After explaining the purpose and constraints of the study, the participants filled in the informed consent form. Three participants in the HIIT group and one in the control group quit before the end of the four-week program for personal reasons.

### Study design

The study design was a randomized controlled trial with a wait-list control group. A researcher who was not involved in the experiment assigned the participants using a computer-based algorithm that generates random numbers, ensuring that each group had an equal chance of being assigned. The experimental group (HIIT) underwent an HIIT regime; the control group (CON) did not receive any training and was instructed to maintain their regular physical activity regimens during the intervention period. After 4 weeks, changes in the selected health-related parameters were compared within and between the two groups. In addition, weekly assessments were conducted to explore the potential benefit made by minimum effort. All participants were required to continue their daily activities and refrain from changing their eating habits during the investigation.

### Training protocol

The HIIT intervention was conducted 3 days per week for 4 weeks in a campus dance classroom. The subjects exercised three times per week, with each training session separated by 48 h (h) to minimize physical and mental exhaustion over the 4-week period. Training sessions were supervised by a female sports science graduate and guided by a female instructor of aerobics for the purpose of HIIT training to be achieved in line with the interval and session effort. The training session consisted of 8 (movements) x 4 repetitions of aerobics separated by 10s of rest (passive recovery) following a progressive training plan as shown in Table 1. All the participants performed a warm-up and cool-down for 5 min each. The aerobic-based HIIT combined dance movements and strength-based exercise (e.g., skipping, front kicks, jumping jacks). Participants wore Polar H7 HR monitors connected to the Polar Team iPad application, monitored by a staff member and displayed on the screen for participants to view during sessions. A target 85% of maximal heart rate (HRmax; 220 − age) or above was demanded to ensure appropriate exercise intensity reached and maintained during the training.

**Table 1:** Description of the 4-week of HIIT intervention.

Week	Sessions	HIIT programs
1 to 4	February 01	4 × 10s, 10s rest
	May 03	4 × (20–25 s), 10s rest
	July 06	4 × (30–35 s), 10s rest
	December 08	4 × 35 s, 10s rest

HIIT, high intensity interval training; s, second.

### Measurements

All the selected health-related parameters were assessed at least 3 days before the start of the intervention and after the final training session. The participants were required to have all the measurements at the laboratory after a minimum 8 h fast and 48 h without engaging in any vigorous activity. The tests were conducted from 8 a.m. to 10 a.m. with the identical sequence in pretest and posttest. Each testing program was executed by the same research staff with standardized operation. HIIT group was provided with the weekly tests (at the end of Week 1 to 3) in the resting day following the training the day before.

### Anthropometrics and body composition assessment

Using a stadiometer and an electronic scale, the subjects’ height and weight were measured to the closest 0.1 cm and 0.1 kg, respectively, while they were wearing light clothes and no footwear. Body mass index (BMI) was calculated by dividing weight (kg) by height (m) squared. Body fat percentage (BF %) were measured using bioelectrical impedance analysis (DBA, 210). Waist circumference (WC) was determined with a soft meter ruler, using the midpoint of the line between the anterior superior iliac crest and the lower edge of the 12th rib as the positioning point and measuring the dimension of the horizontal plane at the end of the participant’s expiratory period. The unit of measurement was cm, accurate to one decimal place. The measurement result was the average value of two measurements with the error not exceeding 1 cm.

### Cardiorespiratory fitness

The Queen’s College step test (QCST), which determines VO2max, was used to evaluate cardiorespiratory fitness. The QCST (r = − 0.75; SEE 2.9 ml · kg^− 1^ ·min^− 1^) developed by McArdle et al. (1972) involved 3 min of continuous walking at a tempo of 88 bpm for women (22 steps/min) on a bench 41.25 cm height. The individuals utilized a four-step cadence of “up-up-down-down” for 3 min in order to maintain a consistent step rate in accordance with a metronome beat. A standing HR was palpated for 15 s during the post-exercise period of 5 to 20 s to conclude the test. The acquired HR was multiplied by 4 to get the beats per minute (bpm), which was then used to estimate the VO2max of female subjects by using the regression equation as follows: VO2max (ml/kg) /min) = 65.81- (0.1847× HR (bpm). The QCST was constructed for college students and has been used effectively in other studies of young adults, including sedentary or non-exercising individuals and overweight or obese female participants (D'Alonzo et al., 2006; Choudhuri et al., 2014; Selland et al., 2021).

### Executive function

To assess executive function, a validated Chinese version of the Stroop color-word test (SCWT) was employed (Cai et al., 2013). SCWT has three subtests: reading colored dots (D), reading non-colored words (W), and reading colored words with different meanings from their real colors (C). The participants were given printed sheets and asked to identify the colors of colored dots, non-colored words, and colored words on them. For each subtest, reaction times (measured in seconds) and accuracy were individually recorded. By deducting the reaction times for colored dots and colored words, the size of the Stroop effect (interference score) was determined. Performance in SCWT was measured using reaction time, accuracy, and interference score.

### Psychological well-being

The Self-Rating Depression Scale (SDS) is a 20-item scale used to assess mood symptoms over the previous 7 days. Each item is graded on a Likert scale from 1 to 4 based on how frequently the symptoms occurred throughout the previous week. The raw score is obtained by adding the scores from each item together, and the standard score is the raw score times 1.25. The Standard Score is divided into four categories: no depression (less than 50); minimum to mild depression (50–59); moderate to severe depression (60–69); and severe depression (more than 70; Cheng et al., 2018; Mao et al., 2019). The survey included the administration of a Chinese SDS. In earlier investigations (Chen et al., 2015), the validity and reliability of the Chinese version of SDS were validated.

### Data analysis

Statistical analyses of all outcomes were conducted using IBM Statistics for Windows (version 26.0) (SPSS, INC 2010, IBM Company, Armonk, NY). An independent-sample t-test was applied to compare the baseline data between the two groups (HIIT and MICT). One-way repeated measures ANOVA with Bonferroni’s post-hoc tests were conducted to evaluate the weekly effect of HIIT exercise on the selected parameters during the 4-week intervention. A two-way mixed analysis of variance (ANOVA) with repeated measures was used to test for main effects of time (pre- vs. post- intervention) and group (HIIT and MICT) as well as interaction of time and group. Partial eta-squared (*ηp^2^*) was used to estimate effect size with the classifications small (0.01), medium (0.06), large (0.14) and very large (2.0; Richardson, 2011). All results were presented as mean ± standard deviation (M ± SD), and *p* values <0.05 were considered significant.

## Results

There were no significant differences on any measured variables between the two groups in the baseline tests (Table 2).

**Table 2:** Baseline data of the groups.

	HIIT (*n* = 23) Mean ± SD	CON (*n* = 25) Mean ± SD	*t*	*P*
Age	20.00 ± 1.48	20.80 ± 1.76	1.70	0.10
Height	165.55 ± 5.63	163.32 ± 5.27	−1.42	0.16
Weight	70.02 ± 8.33	67.46 ± 6.67	−1.18	0.25
BMI	25.53 ± 2.59	25.30 ± 2.21	−0.33	0.74
WC	90.00 ± 6.76	89.70 ± 4.58	−0.18	0.86
BF%	36.23 ± 3.56	35.96 ± 4.43	−0.24	0.81
VO_2_max	40.24 ± 2.98	40.90 ± 2.82	0.79	0.44
SDS score	59.27 ± 3.86	57.50 ± 4.10	−1.54	0.13
Stroop test	14.44 ± 1.60	14.35 ± 1.89	−0.17	0.87

CON, control group; *n*, number; SD, standard deviation; BMI, body mass index, i.e., weight/height^2^ (kg/m^2^); BF %, body fat percentage; WC, waist circumference; VO_2_max, the maximum oxygen uptake; SDS, Self-Rating Depression Scale; *t*, student’s *t*-test; *P*, p value.

### Body composition

**Figure 1:**
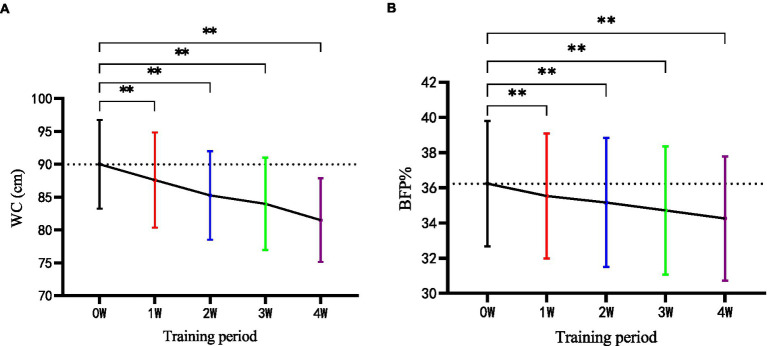
**(A,B)** Weekly measurement of body composition in the HIIT group during the four-week training (WC, waist circumference; BF %, body fat percentage). W, week; kg, kilogram; **p < 0.01.

As shown in Table 3, there were significant changes in the parameters of body composition before and after the 4-week intervention within the group of HIIT, including BF% (*p* < 0.01) and WC (*p* < 0.01). However, no significant change was found in CON. After 4 weeks, the differences between HIIT and CON in WC reached marginal significance (*p* = 0.07). In addition, weekly measurements of HIIT effects showed significant changes in the variables (*p* < 0.01) from the second week when compared with the baseline and maintained the tendency till the end of program (Figures 1A,B).

**Table 3:** Body composition of the two groups before and after the four-week study program.

	HIIT (*n* = 23)		CON (*n* = 25)		ANOVA main effects					
					Group effects		Time effects		Main effects (Time × Group)	
	Pre (0 W)	Post (4 W)	Pre (0 W)	Post (4 W)	*P*	η_P_^2^	*P*	η_P_^2^	*P*	η_P_^2^
**WC**	90.00 ± 6.76	83.55 ± 6.33**	89.70 ± 4.58	89.91 ± 5.23	0.072	0.069	0.000	0.781	0.000	0.802
**BF%**	36.23 ± 3.56	34.25 ± 3.52**	35.96 ± 4.43	36.25 ± 4.54	0.464	0.012	0.000	0.457	0.000	0.606

CON, control group; *n*, number; WC, waist circumference; BF %, body fat percentage; Pre (0 W), pretest before the intervention; Post (4 W), posttest after the fourth week intervention; **P < 0.01, within-group comparison; η_P_^2^, partial eta-squared for effect size.

### Cardiovascular fitness

**Figure 2:**
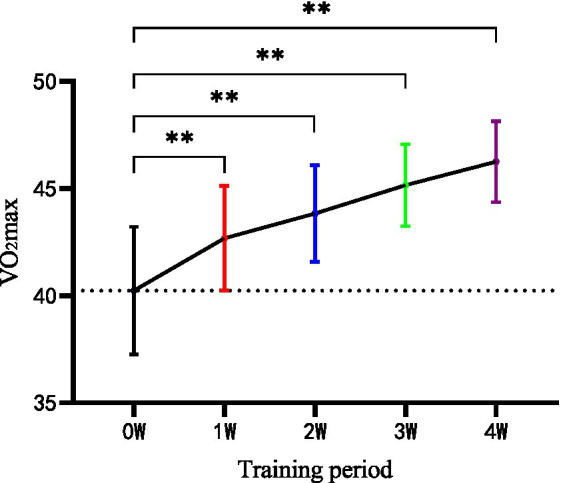
Weekly measurement of maximum oxygen uptake (VO2max) in the HIIT group during the four-week training. W, week; **p < 0.01.

As shown in Table 4, there were significant changes in VO2max before and after the 4-week intervention within the group of HIIT. However, no significant change was found in CON. After 4 weeks, there were significant differences between HIIT and CON in VO2max (*p* < 0.01). In addition, weekly measurements of HIIT effects showed significant change in VO2max (*p* < 0.01) from the second week when compared with the baseline and maintained the tendency till the end of program (Figure 2).

**Table 4:** Cardiovascular fitness of the two groups before and after the four-week program.

	HIIT (*n* = 23)		CON (*n* = 25)		ANOVA main effects					
					Group effects		Time effects		Main effects (Time × Group)	
	Pre (0 W)	Post (4 W)	Pre (0 W)	Post (4 W)	*P*	η_P_^2^	*P*	η_P_^2^	*P*	η_P_^2^
VO2max	40.24 ± 2.98	46.25 ± 1.89**	40.9 ± 2.82	41.31 ± 3.18	0.004	0.167	0.000	0.607	0.000	0.539

CON, control group; n, number; VO2max, the maximum oxygen uptake; Pre (0 W), pretest before the intervention; Post (4 W), posttest after the fourth week intervention; **P < 0.01, within-group comparison, η_P_^2^, partial eta-squared for effect size.

### Psychological well-being

**Figure 3:**
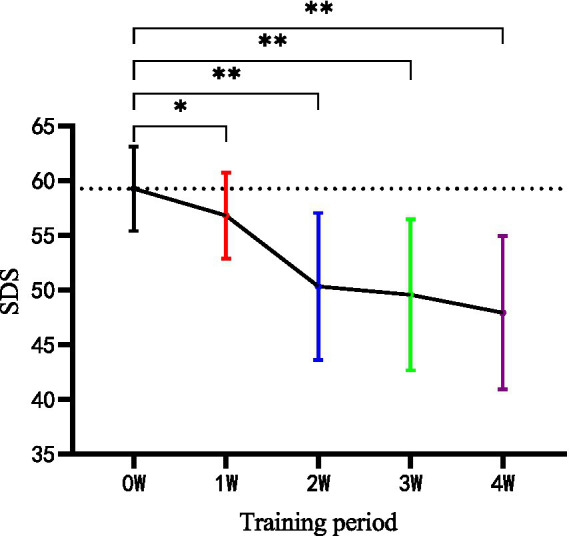
Weekly measurement of SDS scores in the HIIT group during the four-week training. SDS, Self-Rating Depression Scale; W, week; *p < 0.05; **p < 0.01.

As shown in Table 5, there were significant changes in SDS before and after the 4-week intervention within the HIIT group. However, no significant change was found in CON. After 4 weeks, there were significant differences between HIIT and CON in SDS (*p* < 0.05). In addition, weekly measurements of HIIT effects showed significant changes in the variable (*p* < 0.01) from the second week when compared with the baseline and maintained the tendency till the end of program (Figure 3).

**Table 5:** Psychological well-being before and after the four-week study program.

	HIIT (*n* = 23)		CON (*n* = 25)		ANOVA main effects					
					Group effects		Time effects		Main effects (Time × Group)	
	Pre (0 W)	Post (4 W)	Pre (0 W)	Post (4 W)	*P*	η_P_^2^	*P*	η_P_^2^	*P*	η_P_^2^
SDS	59.27 ± 3.86	47.93 ± 7.01**	57.5 ± 4.10	58.95 ± 3.40	0.000	0.268	0.000	0.459	0.000	0.587

CON, control group; n, number; SDS, Self-Rating Depression Scale; Pre (0 W), pretest before the intervention; Post (4 W), posttest after the fourth week intervention; **P < 0.01, within-group comparison; η_P_^2^, partial eta-squared for effect size.

### Executive function

**Figure 4:**
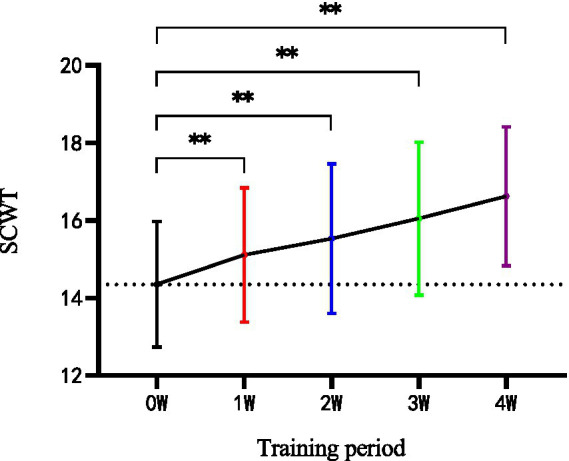
Weekly measurement of SCWT in the HIIT group during the four-week training group. SCWT, Stroop color-word test; W, week; **p < 0.01.

As shown in Table 6, there were significant changes in SCWT before and after the 4-week intervention within the HIIT group. However, no significant change was found in CON. After 4 weeks, there were significant differences between HIIT and CON in SCWT (*p* < 0.01). In addition, weekly measurements of HIIT effects showed significant changes in the variable (*p* < 0.01) from the second week when compared with the baseline and maintained the tendency till the end of program (Figure 4).

**Table 6:** Executive function before and after the four-week study program.

	HIIT (*n* = 23)		CON (*n* = 25)		ANOVA main effects					
					Group effects		Time effects		Main effects (Time × Group)	
	Pre (0 W)	Post (4 W)	Pre (0 W)	Post (4 W)	*P*	η_P_^2^	*P*	η_P_^2^	*P*	**η**_**P**_^**2**^
SCWT	14.44 ± 1.60	16.65 ± 1.83**	14.35 ± 1.89	14.54 ± 1.98	0.035	0.093	0.000	0.572	0.000	0.489

CON, control group; n, number; SCWT, Stroop color-word test; Pre (0 W), pretest before the intervention; Post (4 W), posttest after the fourth week intervention; **P < 0.01, within-group comparison; η_P_^2^, partial eta-squared for effect size.

## Discussion

### Effect of HIIT on body composition

This study showed that 4 weeks of HIIT could effectively reduce the outcomes of body composition including WC and BF% in overweight female young adults. Meanwhile, the study found a significant change after the first week of training, which indicates a quick and effective improvement in the body composition can be induced by HIIT in this target group. A growing body of research has revealed that HIIT could increase fat-free mass and decrease body mass, total or regional fat mass, and WC (Heydari et al., 2012; Hazell et al., 2014). However, with respect to short-term intervention (e.g., 18 sessions for 6 weeks), HIIT interventions demonstrated conflicting results in terms of body composition. These findings include improvement or ineffectiveness (Perry et al., 2008; Metcalfe et al., 2012). The current study showed the effectiveness of HIIT in improving the body composition by weekly measuring during the four-week intervention. Given the conflicting results, more studies are needed to further elucidate the dose–response effect of HIIT on the change in body composition. Regarding the impact of HIIT on fat loss, the following mechanisms have been primarily proposed: changes in metabolism (caused, for example, by hormonal variables), increased excess post-exercise oxygen consumption (EPOC), and adjustments in appetite responses (Martínez-Rodríguez et al., 2021). It is likely that the advantage of HIIT on fat loss may happen in the time following exercise. EPOC explains that the rate of lipolysis and fat oxidation is increased during this phase in a reaction that is exercise intensity-dependent and mediated by beta-adrenergic stimulation, which helps to some extent to replace the relatively low levels of muscle and hepatic glycogen reserves (Richter et al., 1982). These advantages, however, only last for an hour after activity and start to fade (Sevits et al., 2013). Thus, it seems doubtful that EPOC may explain any apparent higher capacity for fat reduction with HIIT (Tucker et al., 2016). While it appears that energy expenditure of the exercise session is crucial to body adiposity reduction, other variables like habitual the eating pattern and physical activity behavior may also contribute to the variations shown among interventions through their effects on energy expenditure. In this study, these factors were not strictly controlled. Therefore, it’s plausible that adjustments to these may have affected how the interventions turned out. Additionally, it has been demonstrated that compensatory processes cause body fat reductions in response to certain exercise dosages to be larger or smaller than anticipated (Rosenkilde et al., 2012). Future research should, it is advised, evaluate the effects of treatments on habitual levels of physical activity, nutrition, and energy expenditure objectively.

### Effect of HIIT on cardiovascular fitness

This study shows that 4 weeks of HIIT can significantly improve the VO2max of overweight young adult women. The previous studies found that different forms of HIIT have been shown to significantly increase VȮ2peak (Heydari et al., 2012; Hazell et al., 2014; Willoughby et al., 2016) and aerobic capacity (Heydari et al., 2012; Hazell et al., 2014). The majority of these studies were lab-based HIIT programs with longer-week duration over 8–10 weeks. Another study by Kong et al. (2016) with five-week Win-gate-based HIIT training in inactive obese young women showed rapid adaptation in cardiovascular function but without statistical significance. In the present study, it was found that four-week HIIT aerobics could result in significant improvement in cardio-vascular function of inactive and overweight young women. The possible reasons might be attributed to the upregulated mitochondrial oxidative enzyme activity (Hazell et al., 2014; Little et al., 2021), the enhanced fractional muscle oxygen extraction (Bailey et al., 2009), and/or the increased stroke volume (Trilk et al., 2011). Additionally, it was found that during the rest periods HIIT can keep the body’s CO, resting ventilation per minute, and oxygen uptake at a high level to improve cardiopulmonary endurance (Nobari et al., 2022). The current study found that HIIT intervention led to a significant negative correlation in the changes between the subjects’ indicators of body composition and maximum oxygen uptake. In light of previous findings, a dose–response relationship of cardiorespiratory fitness adaptations to moderate continuous training was noted in obese individuals (Ohkawara et al., 2007), whereas a similar dose–response effect induced by HIIT was not clear (Logan et al., 2016). Meanwhile, further evidence on dose-effect should be established with regards to various HIIT types, intensities, samples, durance, total volume, etc.

### Impact of HIIT on psychological well-being

In the current research on HIIT’s impact on psychological well-being for participants with a high depressive trend, it was found that 2 weeks of HIIT could reduce the subjects’ SDS scores by 70% and ensure they remain at normal levels for an additional two weeks. Similar results are also found in previous studies. Strassnig et al. (2015) found that HIIT can effectively reduce the symptoms of anxiety and depression among overweight patients with depression and mental disorders. Similarly, Martland et al., showed that HIIT improves the emotional well-being of patients with severe mental disorders (Korman et al., 2020). The results of the current study showed a positive effect of HIIT on depression in overweight female college students. However, the mechanism by which HIIT improves negative emotions remains unknown. Physiological studies proposed that HIIT can reduce tumor necrosis factor α (TNF-α) by inducing expression of the glucocorticoid receptor (GR) in the hippocampus, medial prefrontal cortex (mPFC), and amygdala of CUMS model mice, thus producing an antidepressant effect (Liu et al., 2022). Other evidence suggests that exercise allows individuals to not concentrate on their worries to positively impact on mood regulation (O'Donoghue et al., 2021). Undoubtedly, the study may also support the finding that the improvement in weight loss and body image can reduce social and physical anxiety and enhance self-confidence and the mental health status of obese women (Wu, 2022). In terms of varied indicators and measurements on psychological well-being or ill-being, the effect of HIIT needs to be further explored, especially for the mechanism study.

### Impact of HIIT on executive function

Current knowledge about the impact of HIIT on executive function is limited. Most studies on HIIT have thus focused on its effects on physical health, while the examination of its effects on cognitive ability, including executive function, began relatively recently (Ai et al., 2021). The current study showed that four-week HIIT aerobics significantly improved the results of Stroop test in overweight young adult women. The Stroop test focused on the inhibition of core executive function, suggesting that our low-volume HIIT intervention can improve cognitive ability to some extent, as executive function acts as higher-order cognitive processes. In light of previous studies, the question of whether HIIT affects general executive function or its specific subdomains remains unanswered (Moreau and Chou, 2019). It is also unclear whether specific HIIT characteristics affecting executive function could be investigated because, while the nature of typical exercise characteristics (e.g., exercise duration, exercise mode, and exercise intensity) have been observed to affect executive function (Chang et al., 2012), HIIT contains different exercise features (i.e., working/recovery time ratio and rest interval). Our findings serve as a supplement to prior research as evidence for positive influences on executive function through brief HIIT mode. To our knowledge, it is the first study to conduct HIIT aerobics for overweight female young adults in the ‘real world’ setting. The limited numbers of such studies may be linked to concern over the negative feeling and potential impairment to executive function resulting from high intensity. This belief comes from previous hypotheses that acute exercise of high intensity seems to impair executive function, and were proposed based upon aerobic exercises with continuous rhythms; however, these are different from HIIT, which contains numerous short bouts of high-intensity and rest. It is worth noting, therefore, that the special form of HIIT might result in different effects on executive function.

In summary, this study provides empirical support for the positive effect of HIIT on body composition, cardiovascular fitness, psychological well-being, and executive function in overweight young adult women. This study also assessed the dose-effect relationship of HIIT on these outcomes, providing empirical support for its timeliness. This study has several limitations. First, restricted by the sample size in the current study, it’s hard to draw a conclusive judgment on the efficacy of HIIT in improving the selected health-related parameters. Second, future studies need to carefully manage the influence variables including daily physical activity (intensity and volume), nutrition status (caloric intake) and hormone mediation (e.g., menstrual cycle). Although the non-exercise control group is the strength of this study, absence of its weekly measurement for between-group comparison could lessen methodological rigorousness. Also, it must be noted that there was no other exercising group. Future studies could add and compare other types of physical exercise with the same frequency, duration or volume for further insight into the effects of using HIIT among the target group. Additionally, this study was conducted during the COVID-19 pandemic in addition to the pre-examination weeks before summer vocation, which may have an impact on the emotional status of our subjects to some extent.

## Conclusion

The short-term HIIT aerobics of the campus program conducted in a non-lab setting induced significant improvements in body composition, cardiovascular fitness, psychological well-being and executive function in overweight young adult women. In the future, the minimum training volume and its combination with training intensity in HIIT that could produce the improvement of health outcomes are of interest for further exploration.

## Data availability statement

The raw data supporting the conclusions of this article will be made available by the authors, without undue reservation.

## Ethics statement

The studies involving human participants were reviewed and approved by the Biological and Medical Ethics Committee of Dalian University of Technology. The patients/participants provided their written informed consent to participate in this study.

## Author contributions

LG conceptualized and designed the study, coordinated and supervised data collection, and critically reviewed and revised the manuscript. JC and WY collected data and wrote and revised the manuscript. All authors contributed to the article and approved the submitted version.

## Funding

LG was supported by the Fundamental Research Funds for the Central University, Dalian University of Technology [DUT21RC(3)049], and Liaoning Province Sports Science Association planning project (key project) [2022LTXH067].

## Conflict of interest

The authors declare that the research was conducted in the absence of any commercial or financial relationships that could be construed as a potential conflict of interest.

## Publisher’s note

All claims expressed in this article are solely those of the authors and do not necessarily represent those of their affiliated organizations, or those of the publisher, the editors and the reviewers. Any product that may be evaluated in this article, or claim that may be made by its manufacturer, is not guaranteed or endorsed by the publisher.
